# Supplementary material for: Nuclear speed and cycle length co-vary with local density during syncytial blastoderm formation in a cricket
Source: Nat Commun. 2022 Jul 6;13:3889. doi: 10.1038/s41467-022-31212-8 (PMC9259616; doi:10.1038/s41467-022-31212-8)
Supplement: Supplementary file 3 — Description of Additional Supplementary Files [file 41467_2022_31212_MOESM3_ESM.pdf]

Description of Additional Supplementary Files

File Name: Supplementary Movie 1
Description: *G. bimaculatus* embryo during preblastoderm development, showing that nuclei are not being moved by large-scale, bulk flows of the cytoplasm. Nuclei (white) are labeled with Histone-2B fused to enhanced green fluorescent protein (EGFP). Magenta is the signal from myristoylated and palmitolyated mTomato (mtdT) protein, which is predominantly localized to the cytoplasm in the immediate vicinity of each syncytial nucleus and excluded from putative yolk granules. The field of view was centered 20% of the way from the anterior pole of the embryo, captured during the period of syncytial development when nuclei were initially reaching the anterior pole of the embryo. Dataset was recorded with a confocal microscope, and then 17 consecutive z-slices were combined as a maximal intensity projection.

File Name: Supplementary Software 1
Description: Design files in DXF format for all acrylic components of the constriction device.
